# Ziemissen’s Cyclopædia, Vol. XII

**Published:** 1877-09

**Authors:** 


*Ziemssen's Cyclopædia of the Practice of Medicine.* Vol. XII. Diseases of the Brain and its Membranes. New York: Wm. Wood & Co.

The American reader will find many things that are new and interesting among Ziemssen's volumes. The style of the work is carefully scientific: the treatment of a subject is methodical: comprehensiveneness is aimed at rather than agreeableness in general impression. American writers are more attractive in style; convey a more practical view of disease; they impress the memory with the clinical appearance produced by the patient, and are very sensible, yet suggestive, in therapeutical indications. Many writers in this series of volumes are, we mistrust, in advance of the general practice of the German profession, in discreet conservatism and practical wisdom of treatment. It is a matter of congratulation that we have in the series, the history of disease presented with an accuracy and completeness not before had, while in pathology we have here summarized, the results of the labors of many distinguished foreign investigators, whose authority we are pleased to recognize.

Hermann Nothnagel, Professor of Clinical Medicine and Pathology, at Jena, opens this volume with a section on Cerebral Hemorrhage, Anemia and Hyperemia, which is very good and instructive. The following are from among his statements:

It is an established fact that the amount of blood in the brain is subject to variation. The contents of the skull are practically incompressible by the forces in our organism. The cerebro-spinal fluid furnishes a most important means for regulating the circulation of the brain, receding with the distension of the cerebral vessels, and returning as the distension lessens. The contents of the perivascular lymph spaces act in the same way. The thyroid gland is a further means of regulating intra-cranial circulation. "Maingen was led by a series of investigations to the conclusion that the action of the thyroid gland lies in the fact that when under excessive muscular exertion the veins of the neck are compressed, and there is danger of venous congestion in the brain, this gland becomes swollen and in its turn compresses the carotids, being itself held firmly against the vertebral column by the contraction of the muscles of the neck. In this manner the danger of the further distension of the intra-cranial vessels by way of the carotid is diminished. Guyon confirmed and extended this conclusion."

Venous stasis causes increase of intra-cranial pressure; arterial anemia diminishes it. In hyperemia and anemia there is variation in amount of blood in the intra-cranial cavity or in its pressure. Cerebral anemia may be caused by fatty heart, by aortic valvular disease, and local spasm of arteries may occur in hemicrania and petit mal. Cerebral hyperemia may be caused by thrombosis of the sinuses and by stenosis or obstructions of the larynx.

"Since the observations of Durand-Fardel, the condition designated as état criblé has also been usually regarded as a consequence of long standing hyperemia. This sieve-like appearance of the brain is characterized, as is well known, by the presence on the cut surface of numberless round or oval openings, sometimes as large as a pin's head. These holes were formerly referred entirely to enlargement of the small vessels, but Bizzozero, Golgi, Bastian and others, have proved they depend still oftener upon dilatation of the perivascular lymph sheaths. These dilatations are met with principally in the white substance of the hemispheres, in the coopora striata and in the optic thalami. They are oftenest found at the autopsies of elderly persons, and of those who have suffered from stasis of long duration."

"The most important predisposing cause of cerebral hemorrhage consists in diseases of the cerebral vessels, especially the arteries. In the last twenty-five years this fact has been quite universally recognized, and further, this diseased condition has been supposed to consist essentially in a sclerotic change in the arterial walls. The question was raised, however, how it was in other parts of the body sclerosis of the walls of vessels gives rise to vascular rupture only after aneurisms have formed, whereas in the brain the extravasation seemed to come from vessels without aneurisms."

"Charcot and Bouchard have recently explained this apparent paradox by the demonstration of the so-called miliary aneurisms. These formations had, to be sure, already been observed in single instances, before this time, by Cruveilheir, and Virchow, by whom they were called 'saccular dilatations,' as well as by Heschl, in the pons varolii, and by Meynert. But Charcot and Bouchard were the first to show that the "miliary aneurisms occur *regularly* in the cases of spontaneous hemorrhage, so-called, (out of seventy-seven cases they discovered them in every one,) and that the hemorrhage actually takes place in consequence of the rupture of these formations. Since then their statements have been confirmed by Vulpian, Barth, Behier, Lionville and on the ground of operations extending over several years by Zenker and also by Roth."

"These aneurisms are of reddish color, varying in size from that of scarcely visible bodies, to that of a pins head, and are situated on the arterioles. Their number is very variable, sometimes but few are found, in the vicinity of the ruptured vessel, sometimes they are scattered through the entire brain to the number of one hundred and upwards. The relative frequency of their occurrence in the different regions of the brain is quite constant; their favorite seat is the thalamus opticus and corpus striatum, then the convolutions, pons varolii, centrum ovale, middle cerebellar pedunclesus pedunculi cerebri and medulla oblongata are progressively less apt to be affected."

The symptoms premonitory of an apoplectic attack are described, and the symptomatology of the different lesions as regards their location is well considered. As a conclusion of the subject as to the possibility of localizing hemorrhagic lesions of the cerebral substance, he says:— "We find that this can be done with proximate certainty, *in the presence of certain definite conditions*; only for lesions in: 1, the pons; 2, the pedunculus cerebri; 3, the nucleus lenticularis; 4, the crura cerebeli; and 5, if anesthesia is the only or prominent symptom, for a certain part of the brain which has been specified (occipital or posterior parietal lobe). Furthermore while we are unable to refer lesions to these parts with anything more than approximate certainty, the utmost that we can do for the other regions of the brain, even by dint of the most careful consideration of all the features of the case, is to establish with greater or less certainty a "presumptive diagnosis" and often not even this, especially in cases of large and extensive hemorrhage."

Professor Obernier of Bonn, treats of Tumors of the Brain and its membranes, and gives numerous illustrative cases.

The section on Syphilis of the Brain and nervous system, will be examined with interest by the profession. Professor O. Heubner of Leipzig, the author, states that there are three types of cerebral syphilis generally recognizable, differing in their character, and their progress, and founded on the varying anatomical relation of the lesions present. These are described in summary by him thus:—1. Psychical disturbances, with epilepsy, incomplete paralysis (seldom of the cranial nerves,) and a final comatose condition, usually of short duration. This form occurs where the syphilitic lesion is situate in the cortical portion and adjacent medulla or on the membranes of the brain. 2. Genuine apopletic attacks with succeeding hemiplegia in connection with peculiar somnolent conditions, occurring in often repeated episodes; frequently phenomena of unilateral irritation and generally at the same time irritation of the cerebral nerves. In this class the syphilitic new growth is frequently found, at the base of the brain it projects deeply into the interior, and while spots of softening are usually wanting in the cerebral cortex and in the large mass of medullary white substance (centrum ovale), red or yellow softenings or infarctions, cysts, etc., are regularly found on one of the great ganglia, most frequently in the nucleus lenticularis and nucleus condatus, etc. These internal lesions are very probably consequent upon syphilitic affection of the arteries, which is also a main feature in the pathology of this type of the disease. It is evident that while the paralyses of the cranial nerves in this form of the disease are produced by the exudation at the base of the brain, the hemiplegias are due to the changes in the great ganglia. 3. The course of the cerebral disease is similar to that of dementia paralytica (general paralysis of insane). In all possibility minute processes in the cerebral cortex must be looked upon as underlying these so far mysterious cases. The termination of cerebral syphilis may be, and in fact frequently enough is, a fatal one. But on the other hand when an efficient anti-syphilitic treatment is begun early enough, an absolute or relative cure of the disease, whether it appears in one form or in the other, is decidedly possible.

Acute and chronic Inflammation of the Brain and Membranes are treated of by Professor S. Huguenin, of Zurich. The dura mater is composed of two lamella, one external or periosteal, and another internal layer, and between these are situated the sinuses. The existence of a parietal layer of the arachnoid is denied by all later observers. Pachymeningitis interna hemorrhagica affecting the inner layer of the dura mater is quite frequent. Accounts of the autopsies of insane patients show that it is often present, and it is quite likely to be found with other pathological conditions in the general paralysis of insane. The author gives a minute account of the pathology of the disease, and inclines to the opinion that it is occasioned by rupture of diseased veins near their entrance to the sinuses. Abscess of the brain is fully treated of. The author is very conservative as to interference in treatment of injuries likely to produce intra-cranial suppuration or abscess.

In conclusion, Hypertrophy and Atrophy of the Brain, and a very readable article on Dementia Paralytica, or general paralysis of insane, is given by Prof. E. Hitzig, of Zurich, whose investigations in the localization of the functions of the brain are widely known. W.

